# Research trends in total knee arthroplasty in India: a five-year bibliometric analysis

**DOI:** 10.1007/s00402-026-06488-w

**Published:** 2026-10-07

**Authors:** Saidan Shetty, Sandeep Vijayan, K. G. Mohandas Rao, Sharath K. Rao, Yogendra Nayak, Bincy M. George

**Affiliations:** 1 Department of Orthopaedics, Kasturba Medical College, Manipal Academy of Higher Education, Manipal, India; 2 Department of Pharmacology, Manipal College of Pharmaceutical Sciences, Manipal Academy of Higher Education, Manipal, India; 3 Department of Physiotherapy, Manipal College of Health Professions, Manipal Academy of Higher Education, Manipal, India; 4 Department of Basic Medical Sciences, Manipal Academy of Higher Education, Manipal, India

**Keywords:** Total knee arthroplasty, Bibliometric analysis, India, Orthopaedics, Research trends

## Abstract

**Background:**

Total Knee Arthroplasty (TKA) is one of the fastest-growing orthopaedic procedures in India, driven by demographic transitions, increased life expectancy, and the rising burden of knee osteoarthritis. With expanding procedural volumes, periodic evaluation of scientific output is essential to identify evolving research priorities, institutional leadership, and collaborative patterns. This study aimed to perform a bibliometric analysis of Indian TKA research published over the past five years.

**Methods:**

Publications were retrieved from the Scopus database using a structured search strategy for TKA-related terms. The search was restricted to studies published between 1 January 2021 and 30 December 2025 and conducted in an Indian setting. Bibliometric indicators assessed publication trends, document types, journals, authorship, institutional productivity, and state-wise contributions. Network-based bibliometric visualizations were generated using VOSviewer.

**Results:**

A total of 860 publications were identified, showing a steady increase in research output with peak productivity in 2025 (*n* = 221). Maharashtra and Delhi were the leading contributors, and the All-India Institute of Medical Sciences was the most productive institution. Original research articles accounted for 69.5% of publications. Emerging themes included robotic-assisted surgery, AI-based modeling, and advanced rehabilitation. International collaboration was observed in 24.18% of studies, mainly with the UK and USA.

**Conclusion:**

Indian TKA research has demonstrated substantial growth and increasing thematic maturity over the past five years, with a shift toward technology-driven and collaborative research. Strengthening national registries, encouraging multicentric collaboration, and expanding research beyond metropolitan centers are essential to sustain future impact.

**Trial registration:**

Not applicable.

**Supplementary Information:**

The online version contains supplementary material available at https://doi.org/10.1007/s00402-026-06488-w.

## Background

Total Knee Arthroplasty (TKA) is widely regarded as the definitive surgical intervention for end-stage knee osteoarthritis and other debilitating knee conditions [1–3]. Globally, the demand for TKA has increased substantially over the past two decades, driven by population ageing, rising obesity rates, higher patient expectations, and continual improvements in implant design and surgical techniques [4, 5]. India has paralleled global growth patterns and experienced a substantial increase in TKA utilization, thereby, increasing life expectancy, and expanding access to advanced orthopaedic care [6, 7].

The last five years represent a particularly critical period for knee arthroplasty research in India. This timeframe coincides with the accelerated adoption of enhanced recovery protocols, widespread introduction of robotic-assisted and computer-navigated TKA, growing engagement of private-sector hospitals in academic research, and increasing international collaboration [8, 9]. Furthermore, the COVID-19 pandemic and its aftermath have reshaped elective surgical practice, clinical research priorities, and health-system resilience, making recent literature especially reflective of contemporary clinical realities [10–12].

Scientific research plays a central role in guiding evidence-based arthroplasty practice by evaluating outcomes, refining surgical techniques, assessing implant performance, and optimising rehabilitation strategies [13, 14]. Bibliometric analysis offers a systematic and quantitative approach to evaluate the evolution, impact, and collaborative structure of research within a defined field [15, 16]. Recent bibliometric explorations have demonstrated the utility of this method in mapping the fragmented landscape of navigated and robotic-assisted arthroplasty, emphasizing how such analyses identify the transition from experimental technology to established clinical practice [17]. Furthermore, the evolution of knee-related research is increasingly characterized by a shift toward biological repair and the integration of biomechanical feedback, reflecting a global drive toward precision medicine and personalized rehabilitation in joint care [18, 19].

Previous scientometric studies have assessed orthopaedic and arthroplasty-related research output from India over extended periods [20, 21]; however, focused analyses specifically examining recent five-year trends in TKA research remain limited. This specific temporal focus is necessary because the last half-decade represents a distinct “inflection point” in Indian orthopaedics that longitudinal studies spanning 20 years often dilute. By isolating the 2021–2025 period, several unique insights emerge that are otherwise obscured in long-term analyses. First, this window captures the “maturation phase” of robotic and digital surgery in India, moving beyond early adoption reports to outcome-based studies. Second, it reflects the “Post-COVID Academic Pivot,” revealing how Indian researchers adapted to the temporary cessation of elective surgeries by shifting focus toward telemedicine, telerehabilitation, and health economics. Third, a focused five-year analysis uniquely highlights the rising academic prominence of high-volume private corporate hospitals, which are now challenging the traditional research dominance of government-funded medical colleges [19, 21, 22].

Given the rapid technological and clinical evolution of knee arthroplasty, an updated bibliometric evaluation centred on the most recent literature is essential. Analysing the past five years of Indian TKA research provides insight into contemporary research priorities such as the transition from implant-centric research to patient-reported outcome measures and cost-utility analyses. It further identifies institutional leadership and existing gaps that may inform future policy, funding, and collaborative strategies. Accordingly, the present study aims to provide a comprehensive bibliometric analysis of Indian TKA research published from 1 January 2021 till 30 December 2025.

## Materials and methods

### Study design and data source

This bibliometric study was designed and reported in accordance with established methodological frameworks commonly adopted for scientometric analyses in orthopaedics and arthroplasty research, drawing on best practices described in recent bibliometric literature [23–26]. The Scopus database (https://www.scopus.com) was selected as the data source due to its broad coverage of peer-reviewed biomedical and orthopaedic journals and the availability of built-in analytical tools for bibliometric evaluation.

### Search strategy and eligibility criteria

A structured search strategy was applied using relevant keywords and synonymous terms such as “total knee arthroplasty” OR “TKA” OR “total knee replacement arthroplasty” OR “total knee joint replacement” OR “knee replacement total” OR “total knee replacement” OR “knee replacement*” OR “TKR” OR “knee arthroplasty total” OR “knee arthroplast*” to the title, abstract, and keyword fields. The search was restricted to documents published from 1 January 2021 till 30 December 2025 and limited to publications with an Indian study setting. All document types indexed in Scopus, including original research articles, review articles, conference papers, and letters, were included.

The complete Scopus search strategy, including the exact search query, search date, filters applied, and the number of records retrieved at each stage of the screening process, is provided in Supplementary File 1. Studies were initially identified using the Scopus country filter and subsequently cross-verified through manual screening of author affiliations and publication details to confirm their relevance to the Indian setting.

### Data extraction and analysis

Bibliographic data were exported from the Scopus database for analysis. The extracted variables included year of publication, authorship information, institutional affiliations, geographic location, document type, and source journal. For institutional and state-wise analyses, author affiliation data exported from Scopus were used. In publications involving multiple institutions or multiple Indian states, each unique institution and state represented within a publication was counted once to reflect collaborative research contributions, while duplicate affiliations within the same publication were excluded from repeated counting.

Descriptive bibliometric indicators available within the Scopus database were initially utilised to examine publication distribution and key bibliometric indicators. Subsequently, the extracted data were analysed using spreadsheet-based methods to evaluate annual publication trends, authorship patterns, journal distribution, institutional productivity, and geographic contribution. Collaborative relationships among authors, keywords, citations, and countries were explored using network-mapping techniques implemented in VOSviewer (version 1.6.20).

## Results

### Overview of retrieved literature

A total of 73,170 TKA-related documents were identified globally, of which 1,710 originated from India. After restricting the timeframe from 1 January 2021 till 30 December 2025, 860 Indian publications were included in the final analysis.

### Temporal trends in Indian publication output

**Fig. 1:**
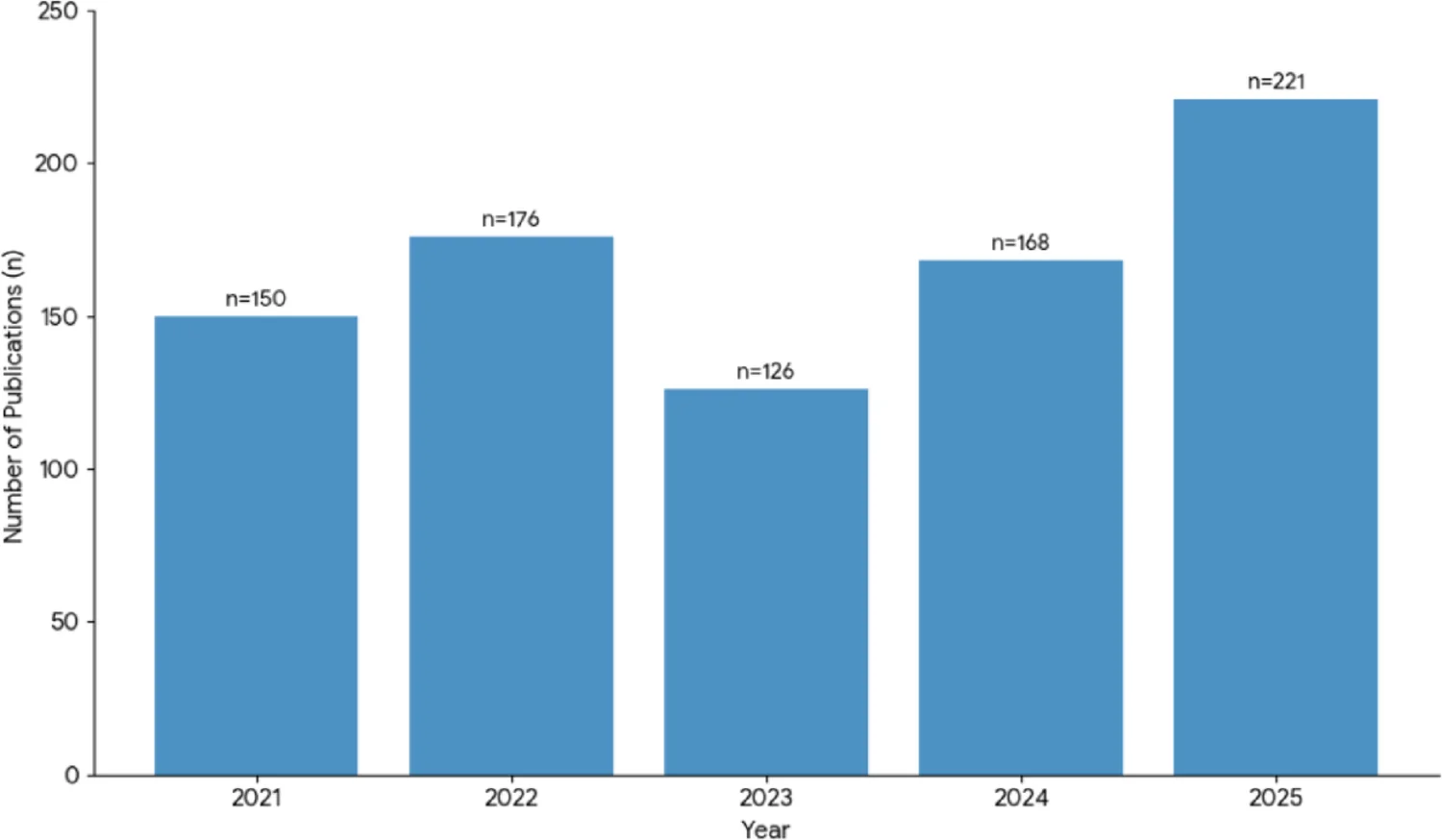
Annual publication output of total knee arthroplasty (TKA) research in India from 2021 to 2025

The temporal distribution of TKA-related publications from India shows a fluctuating but ultimately upward trajectory over the analyzed period. Publication output increased from 150 articles in 2021 to 176 in 2022, followed by a temporary decline in 2023 (*n* = 126). Research activity rebounded in 2024 (*n* = 168) and peaked in 2025 with 221 publications. The most prolific year was 2025, representing the highest volume of research and suggesting a surge in clinical and academic interest in knee arthroplasty within the Indian medical community. Additionally, 19 articles that were accepted in 2025 were identified as ahead-of-print publications for 2026, reflecting the most recent advancements in the field. The yearly distribution of the number of publications in TKA research in India is depicted in Fig. 1.

### Growth trends in Indian publications on TKA from inception till present

The longitudinal analysis of 1,710 TKA-related documents from India reveals a distinct evolutionary trajectory spanning over three decades, starting from the first recorded publication in 1990. The research landscape is characterized by three developmental phases. The foundational phase (1990–2000) was marked by sporadic academic output, with several years specifically 1991, 1993, 1994, and 1996, recording zero publications. During this decade, the total output remained minimal, reflecting the early stages of arthroplasty adoption in the country.

**Fig. 2:**
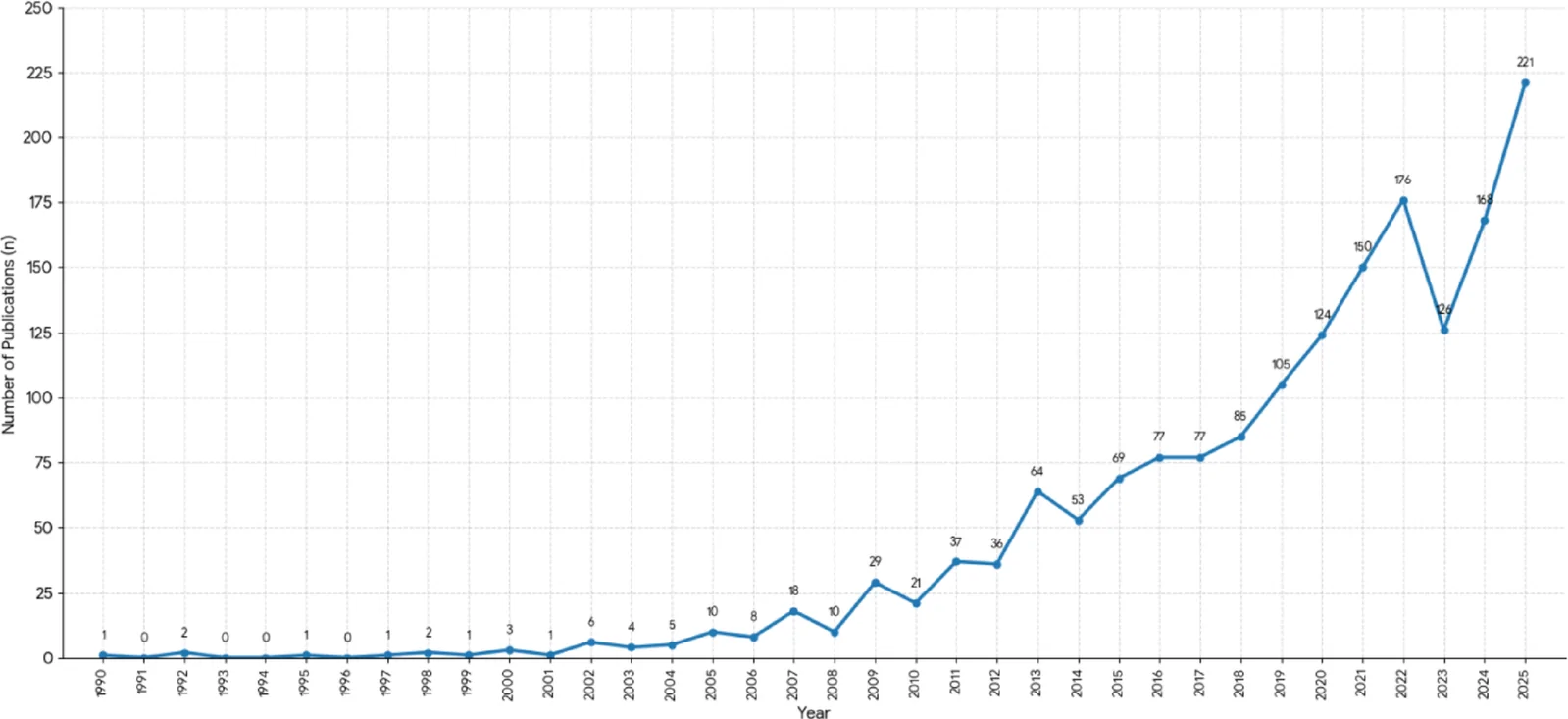
Line graph depicting the annual publication output of TKA research in India from inception to 2025. The line graph illustrates the progressive growth in publication output over time

The steady expansion phase (2001–2015) saw the field achieve year-on-year consistency. During this period, publication volume began to climb steadily as specialized arthroplasty units were established in major metropolitan hospitals. A significant milestone was reached in 2013, when annual output first surpassed 50 articles (*n* = 64). The acceleration phase (2016–2025) represents the most prolific era in the history of Indian TKA research, accounting for the vast majority of cumulative literature. Annual publications exceeded the 100-article threshold for the first time in 2019 (*n* = 105). The most recent five-year window (2021–2025) contributed increasing publications, representing 49.2% of the total historical output. Within this window, research output grew from 150 articles in 2021 to an all-time peak of 221 in 2025, the first instance of India exceeding 200 publications in a single year. Despite a transient decline in 2023 (*n* = 126), likely due to post-pandemic elective surgery disruptions, the rapid recovery in 2024 and the record-high volume in 2025 demonstrate a robust academic momentum. Early data for 2026 (*n* = 19) further indicates that this upward trend is sustained (Fig. 2).

### Authorship patterns

**Fig. 3:**
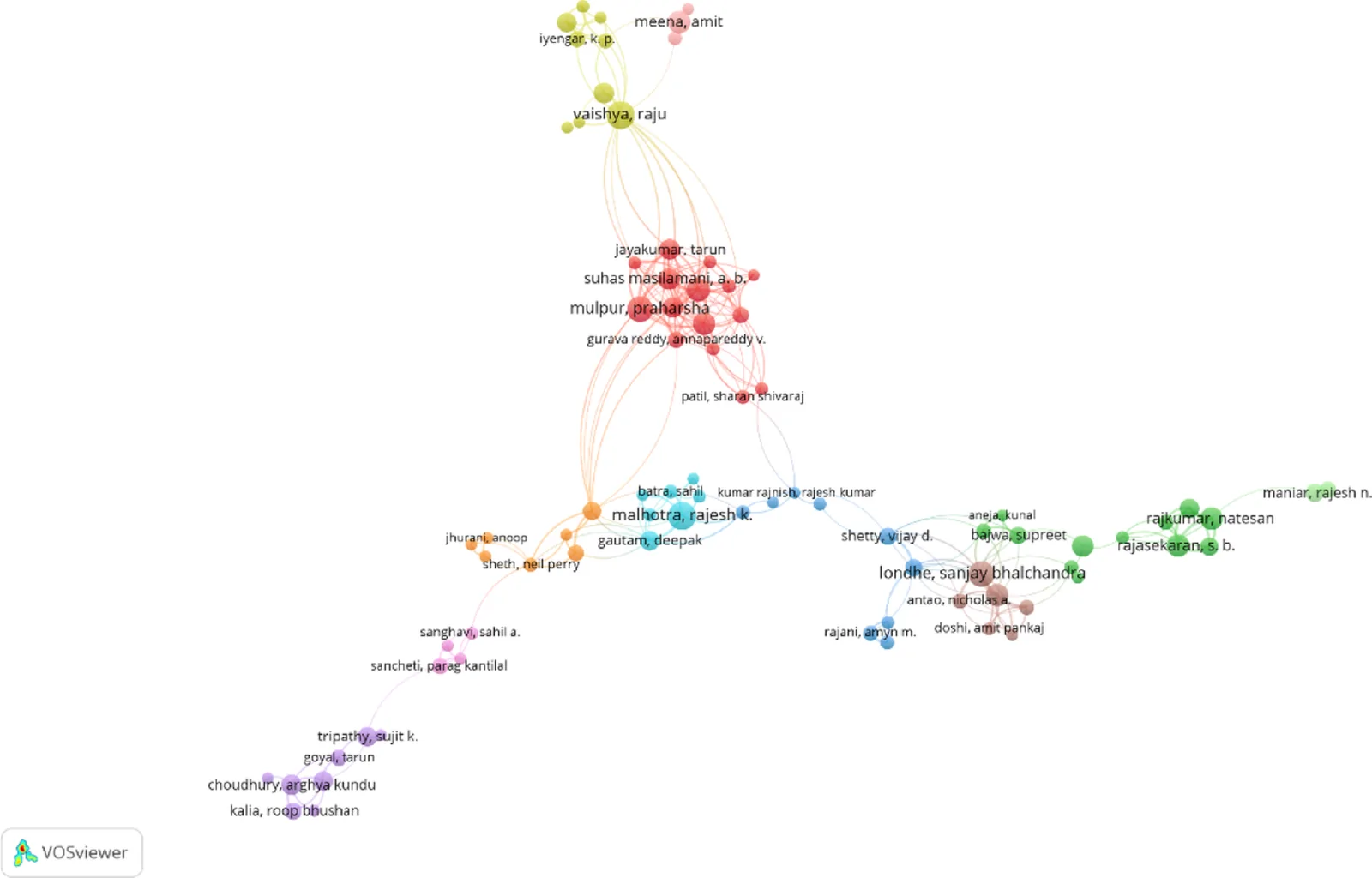
Network visualization of co-authorship in Indian TKA research generated using VOSviewer. The analysis includes authors with a minimum of five publications. Each node represents an author, with larger nodes indicating greater publication output. Links represent co-authorship relationships, and different colours denote distinct author collaboration clusters

Most publications were multi-authored, highlighting collaborative research practices. An analysis of authorship patterns reveals an average of approximately five authors per paper (mean = 4.96). Most studies involved mid-sized teams of four to six authors (*n* = 439), followed by two- to three-author collaborations (*n* = 243). Larger collaborative groups of seven to ten authors contributed substantially (*n* = 135), while single-author publications were uncommon (*n* = 28). A small proportion of studies (*n* = 15), involved extensive multi-institutional collaborations exceeding ten authors, underscoring increasing research networking within the field. This distribution underscores the collective nature of contemporary research in India, where multi-authored studies are the standard practice (Fig. 3).

### State-wise distribution of publications

**Fig. 4:**
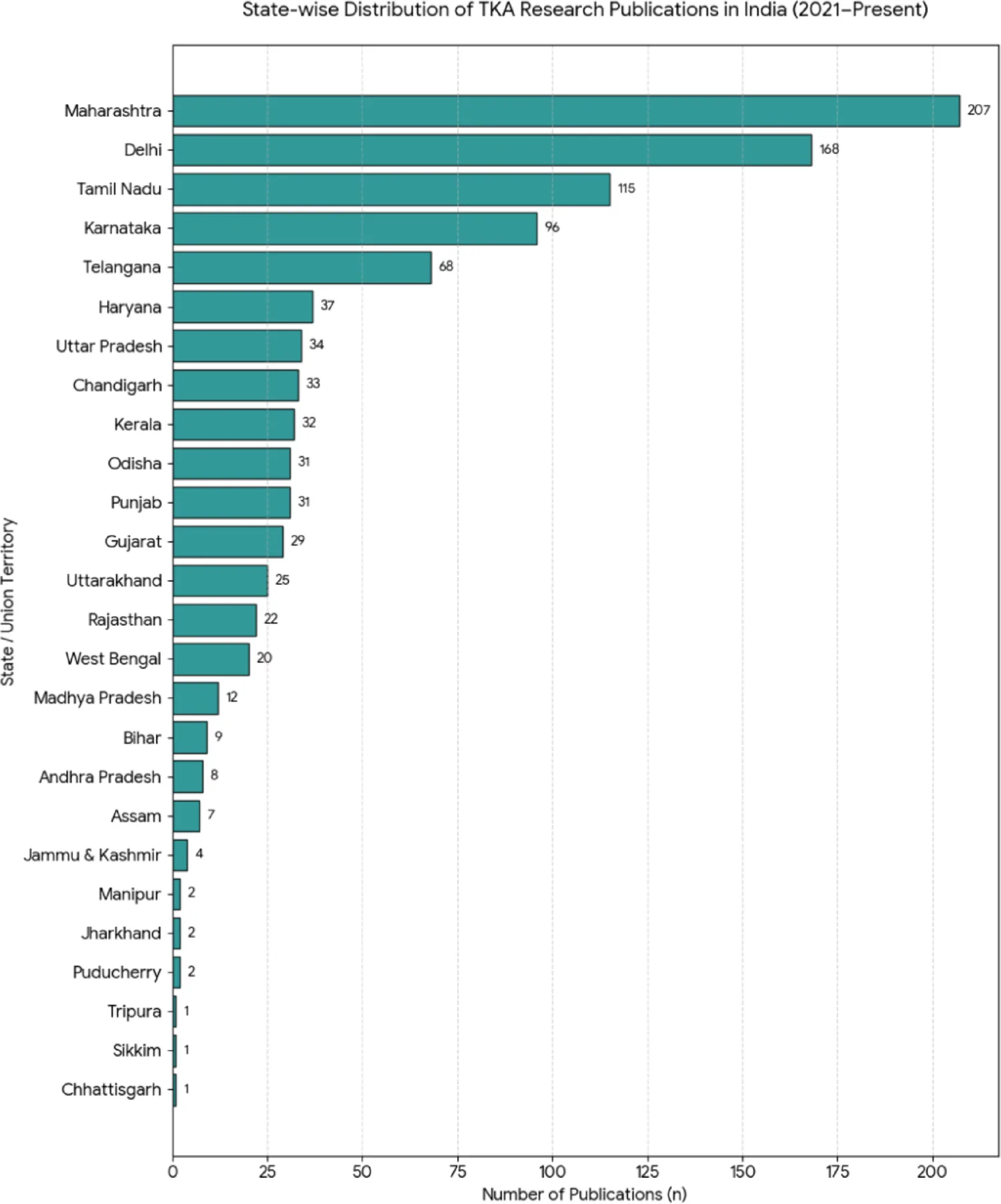
State-wise distribution of TKA research publications in India from 2021 to 2025. Publication counts were determined through manual screening of the study settings reported in each article

Research output was geographically concentrated in major academic and metropolitan regions. Maharashtra (*n* = 207) and Delhi (*n* = 168) were the leading contributors, followed by Tamil Nadu (*n* = 114), Karnataka (*n* = 95), and Telangana (*n* = 68). Several other states, including Chandigarh (*n* = 33) and Kerala (*n* = 32), demonstrated moderate output, while smaller and northeastern states contributed fewer publications. This distribution highlights a research landscape predominantly led by regions with established medical and technical institutions (Fig. 4).

### Institutional contributions

**Fig. 5:**
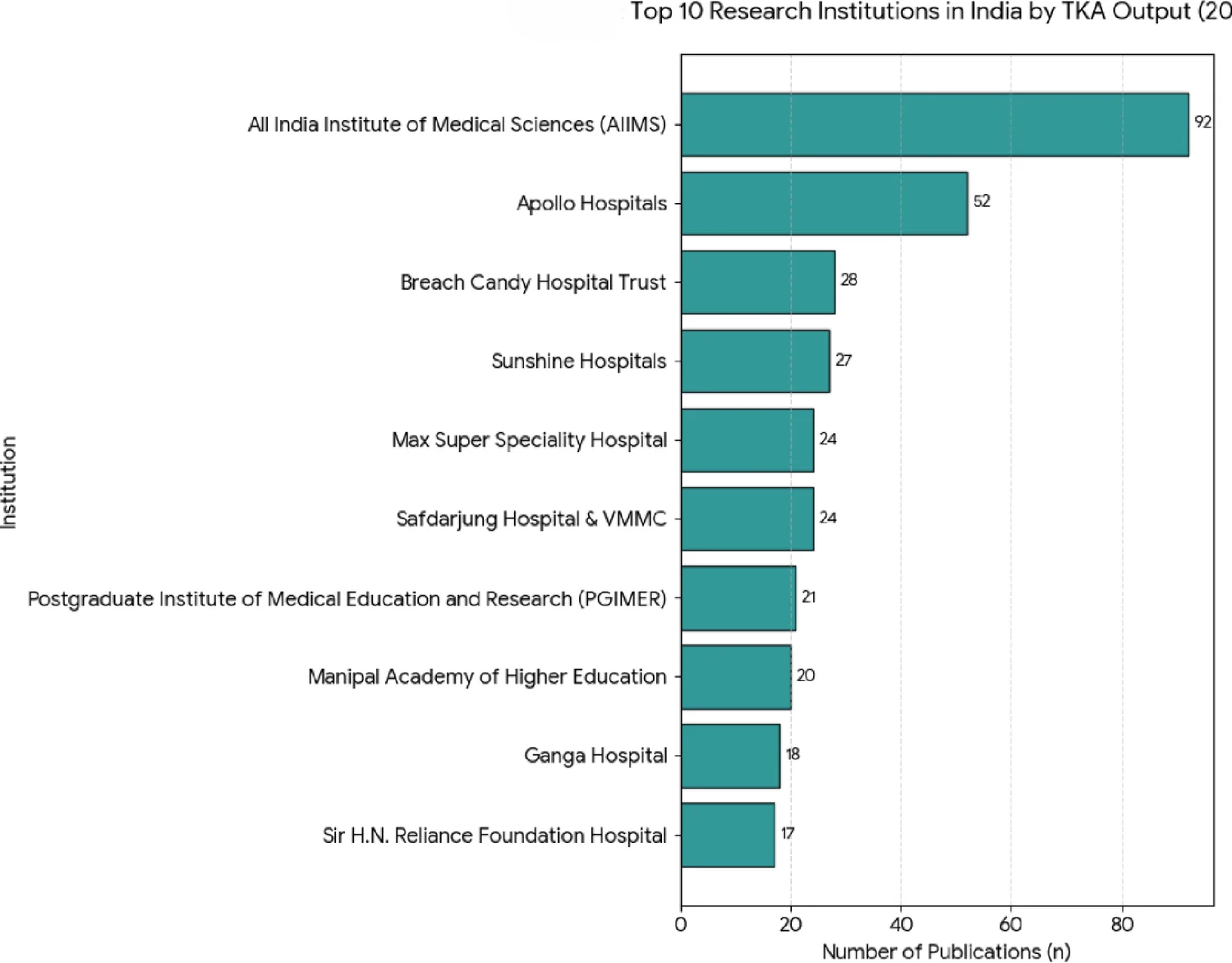
Institutional contributions to TKA research in India from 2021 to 2025. The bar chart presents the top 10 institutions ranked by the number of publications, highlighting the leading contributors to Indian TKA research

Leading tertiary care hospitals and academic institutions dominated the publication output. The top 10 highest-contributing research institutions and hospitals in India demonstrate a mix of public academic centers and premier private healthcare providers. The All India Institute of Medical Sciences (*n* = 92) leads as the most prolific research entity, followed by the Apollo Hospitals group (*n* = 52). Significant research contributions also originate from specialized private institutions such as Breach Candy Hospital Trust (*n* = 28) and Sunshine Hospitals (*n* = 27). The Safdarjung Hospital & Vardhman Mahavir Medical College (*n* = 24) and Max Super Speciality Hospital (*n* = 24) are also leading centers for research. The Postgraduate Institute of Medical Education and Research (PGIMER) in Chandigarh (*n* = 21) and the Manipal Academy of Higher Education (*n* = 20) remain major hubs for academic excellence. Rounding out the top ten are Ganga Hospital (*n* = 18) and the Sir H.N. Reliance Foundation Hospital (*n* = 17), reflecting a diverse and balanced landscape of clinical and academic excellence in Indian medical research (Fig. 5).

### Journals and research dissemination

**Fig. 6:**
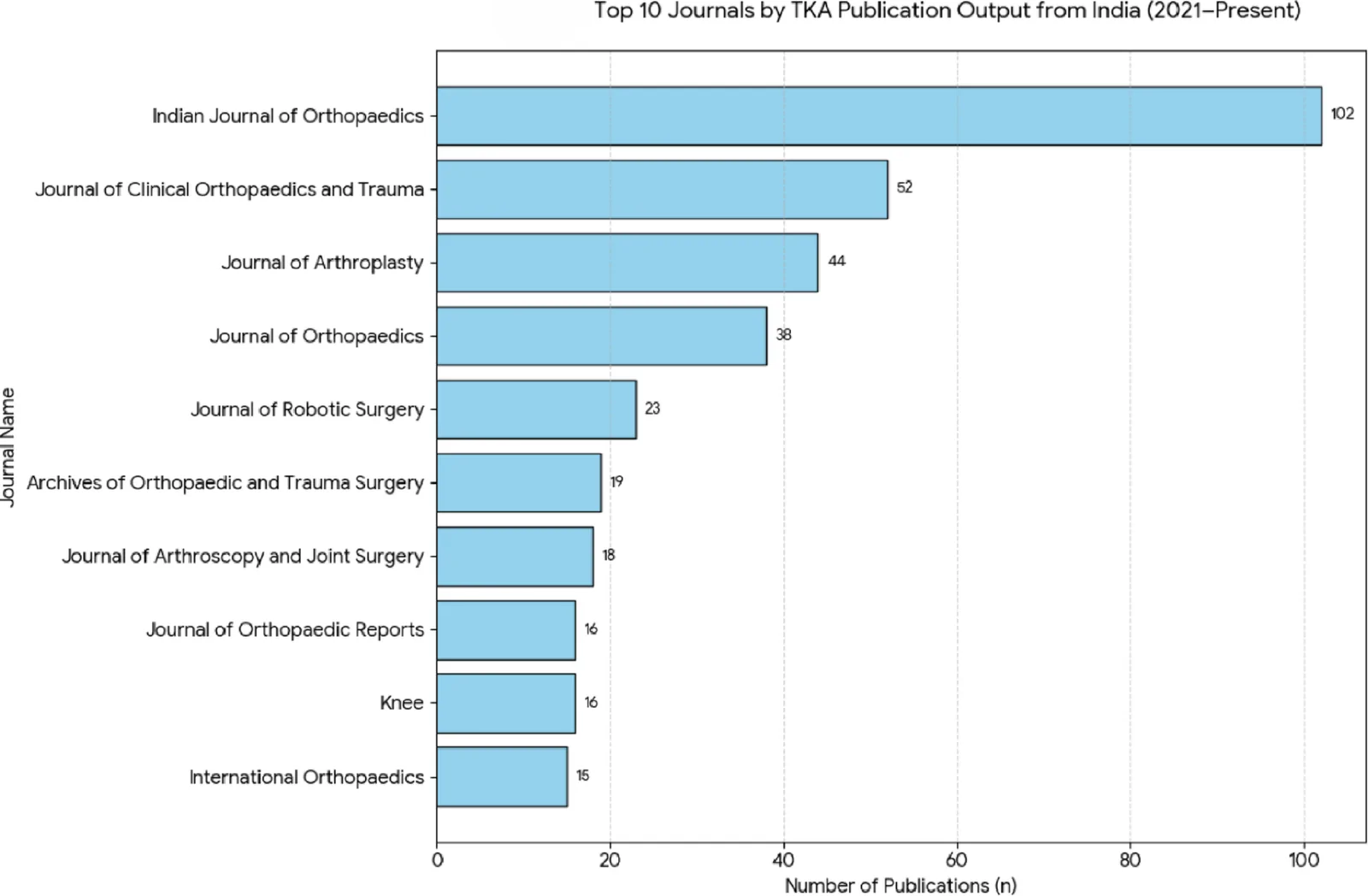
Publication output of the top 10 journals publishing Indian TKA research from 2021 to 2025. Bars represent the number of publications contributed by each journal

The scholarly dissemination of Indian research on TKA is distributed across a variety of specialized orthopaedic and surgical publications, with a strong emphasis on both domestic and international high-impact journals. The Indian Journal of Orthopaedics (*n* = 102) stands as the primary vehicle for publication, followed by the Journal of Clinical Orthopaedics and Trauma (*n* = 52). The Journal of Arthroplasty (*n* = 44) and the Journal of Orthopaedics (*n* = 38) also feature heavily in the dataset, underscoring the global relevance of Indian clinical data. Other significant contributors include the Journal of Robotic Surgery (*n* = 23), reflecting a growing interest in technology-assisted procedures, as well as the Archives of Orthopaedic and Trauma Surgery (*n* = 19) and the Journal of Arthroscopy and Joint Surgery (*n* = 18). Rounding out the top ten are The Knee (*n* = 16), Journal of Orthopaedic Reports (*n* = 16), and International Orthopaedics (*n* = 15). Collectively, these top ten journals account for a substantial portion of the research output, providing a robust platform for the exchange of surgical techniques, clinical outcomes, and technological advancements in the field (Fig. 6).

### Open access journals

The accessibility of TKA-related research from India demonstrates a balanced distribution between open-access and subscription-based models, reflecting a diverse dissemination strategy. Of the 860 publications analyzed, 52.7% (*n* = 453) are available through Open Access channels, while 47.3% (*n* = 407) are restricted to Subscription or pay-walled access. Within the open-access subset, Gold Open Access is the most prevalent route (*n* = 231), where research is made immediately and permanently free at the point of publication. A portion of the literature also utilizes Green Open Access (*n* = 312 including overlaps), indicating a strong practice of self-archiving in institutional or subject-specific repositories. Other models, such as Hybrid (*n* = 35) and Bronze (*n* = 40) access, contribute to a smaller share of the output.

### Thematic areas in TKA research

The thematic landscape of TKA research in India is characterized by a strong surgical core complemented by a burgeoning interest in technological integration and post-operative care. The majority of research falls under the subdomain of Orthopaedics and Surgery (*n* = 603), focusing on clinical outcomes, prosthetic design, and traditional versus minimally invasive surgical techniques. A significant and rapidly expanding secondary domain is Engineering and Technology-related publications (*n* = 98), which explores the utility of robotic-assisted surgery, artificial intelligence for predictive modeling, and computer-navigated systems to enhance surgical precision. Additionally, a focused segment of the literature is dedicated to Rehabilitation and Allied Health (*n* = 56), highlighting the critical role of specialized physiotherapy protocols, gait analysis, and home-based recovery in optimizing long-term patient mobility. The remaining output (*n* = 103) spans General Medicine and Multi-disciplinary areas, addressing systemic comorbidities and public health implications of knee arthroplasty. This distribution underscores a shift in the Indian research paradigm from purely clinical observations toward a sophisticated, multi-disciplinary approach that integrates robotic technology and advanced rehabilitation sciences.

### Type of studies

The TKA-related publications from India exhibit a diverse range of study types, reflecting a comprehensive approach to scientific inquiry that spans from primary clinical investigations to high-level evidence syntheses. Original research articles (*n* = 589) form the backbone of the dataset, accounting for nearly 69.5% of the total output and representing the high volume of primary clinical trials, observational studies, and surgical outcome reports. Review articles (*n* = 101) comprise the second-largest category, providing essential summaries of existing literature and expert perspectives on evolving trends. The academic breadth is further enhanced by book chapters (*n* = 58) and letters to the editor (*n* = 49). Other document types include conference papers (*n* = 31), which capture early-stage research presented at international forums, as well as editorials (*n* = 12) and short notes. This distribution indicates a mature research landscape where primary data collection is balanced by significant efforts in academic synthesis and professional discourse.

### Keywords in TKA research

The distribution of keywords across the identified subdomains underscores the multifaceted nature of Indian research in TKA. Within the Orthopaedics and Surgery cluster (*n* = 603), the literature is primarily centered on established clinical concepts, with “total knee arthroplasty” (*n* = 458), “knee osteoarthritis” (*n* = 166), and “knee replacement” (*n* = 164) representing the most frequently occurring keywords. These high-frequency terms form the core of Indian TKA research and reflect the longstanding emphasis on degenerative joint disease management and surgical intervention.

The Engineering and Technology cluster (*n* = 98) highlights the increasing integration of advanced surgical technologies into TKA research. Frequently occurring keywords include “robotic-assisted surgery” (*n* = 44), “robotic surgery,” “computer navigation,” and “navigation,” reflecting growing interest in precision surgery and technology-assisted procedures. The Rehabilitation and Allied Health cluster (*n* = 56) focuses on patient-centered recovery, with keywords such as “quality of life” (*n* = 21), “functional outcomes” (*n* = 20), “rehabilitation,” and “range of motion,” emphasizing long-term postoperative function and quality of care. The General Medicine and Others category (*n* = 103) encompasses broader clinical themes including infection, analgesia, systematic reviews, and perioperative management, serving as a bridge between orthopaedic practice and multidisciplinary healthcare research.

**Fig. 7:**
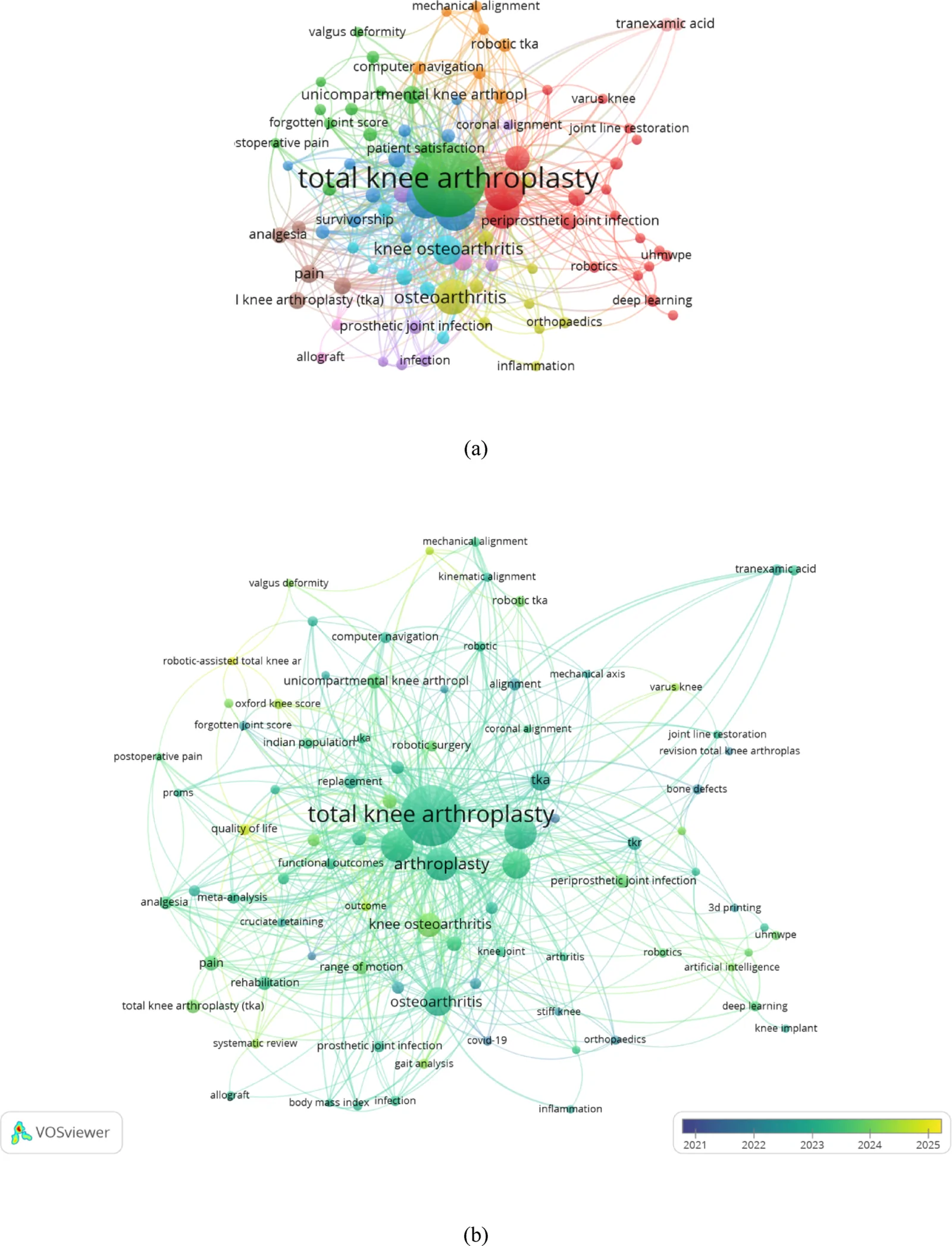
**a** Network visualization of keyword co-occurrence in Indian TKA research generated using VOSviewer. The analysis includes keywords with a minimum occurrence of five. Node size is proportional to keyword frequency, links represent co-occurrence relationships, and colours indicate distinct thematic clusters. **b** Overlay visualization of keyword co-occurrence in Indian TKA research generated using VOSviewer. Keywords with a minimum occurrence of five were included. Node size represents keyword frequency, links indicate co-occurrence relationships, and the colour gradient represents the average publication year of each keyword, ranging from earlier topics (blue; 2021–2022) to more recent topics (yellow; 2024–2025), illustrating the temporal evolution of research themes

Figure 7a depicts the network visualization and Fig. 7b represents the overlay visualization (publication year) of co-occurrence of all keywords in Indian TKA research in the past five years. The overlay visualization (Fig. 7b), generated using the average publication year of keywords, further demonstrates the temporal evolution of research themes over the five-year study period. Earlier research (predominantly 2021–2022; blue to cyan nodes) focused on conventional topics such as osteoarthritis, prosthetic joint infection, gait analysis, orthopaedics, rehabilitation, and pain management. Keywords with intermediate average publication years (2023–2024; green nodes) included robotic surgery, computer navigation, alignment techniques, quality of life, and functional outcomes, indicating a transition toward technology-assisted surgery and patient-centered outcome assessment. The most recent keywords (2024–2025; yellow-green nodes) include artificial intelligence, deep learning, robotic-assisted TKA, kinematic alignment, mechanical alignment, valgus deformity, varus knee, and joint line restoration, demonstrating a clear shift toward digital orthopaedics, intelligent surgical planning, and precision medicine. This temporal progression objectively supports the emergence of AI-, robotics-, and technology-driven research as contemporary themes in Indian TKA literature. Collectively, these findings illustrate that Indian TKA research has evolved from predominantly clinical and surgical investigations toward multidisciplinary research integrating engineering innovations, digital technologies, and comprehensive rehabilitation strategies.

#### High-cited authors and original papers

**Fig. 8:**
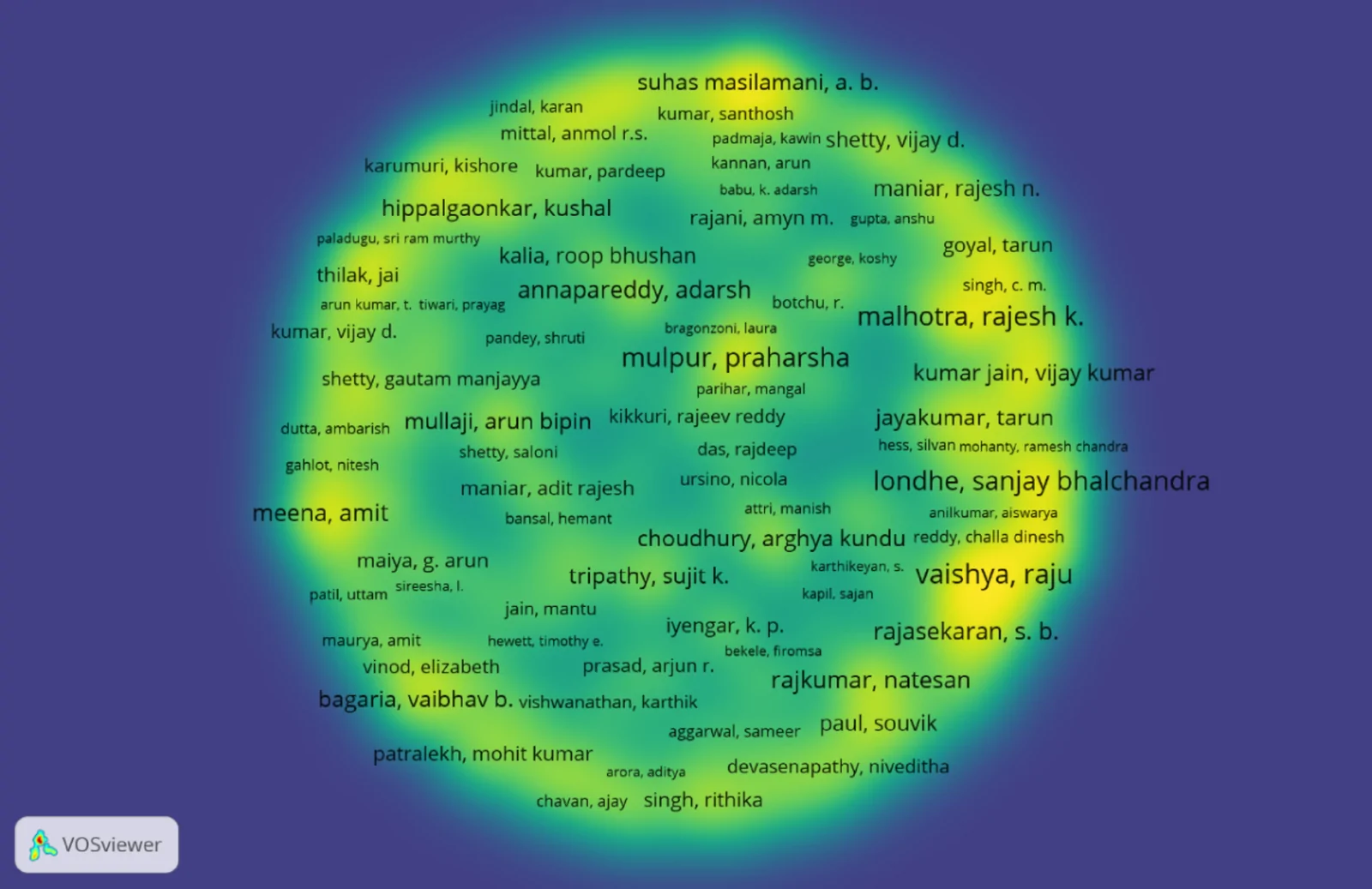
Density visualization of author citations in Indian TKA research generated using VOSviewer. The analysis includes authors with a minimum of five citations. Regions with warmer colours (yellow) indicate areas of higher citation density, whereas cooler colours (green to blue) represent lower citation density, highlighting the most influential authors within the citation network

The impact of individual researchers within the Indian TKA landscape is characterized by high-volume contributors with sustained clinical influence and leaders of landmark original investigations. Singh, S. (*n* = 229 citations) and Sharma, S. (*n* = 215) emerge as the authors with the highest cumulative citation impact in the 2021–2025 period, driven by a high volume of peer-recognized clinical reports. They are followed closely by Vaishya, R. (*n* = 212 citations) and Meena, A. (*n* = 196 citations), whose prolific output across multiple sub-domains of arthroplasty has established them as central figures in the national research network. Notably, Malhotra, R. (*n* = 128 citations) represents academic leadership from premier public institutions like AIIMS, while researchers such as Mulpur, P. (*n* = 149 citations), Mullaji, A.B. (*n* = 145 citations), and Vaidya, N. (*n* = 141 citations) represent the vanguard of innovative private centers specializing in robotic integration and complex primary surgery. Figure 8 illustrates the citation network of prominent authors in the Indian TKA research landscape, specifically mapping individuals with a minimum threshold of five citations. The Top 5 highly cited original research papers are presented in Table 1, representing the most influential primary data coming out of India.

**Table 1:** Bibliometric profile of top five highly cited papers in TKA research in India in the past five years

Rank	Lead author	Journal	Title	Year	Citations
1	Kumar, R.	Materials Today: Proceedings	Multiple-criteria decision‐making and sensitivity analysis for selection of materials for knee implant femoral component	2021	90
2	Vaidya, N.	Journal of Robotic Surgery	Robotic-assisted TKA leads to a better prosthesis alignment and a better joint line restoration as compared to conventional TKA: a prospective randomized controlled trial	2022	87
3	Mulpur, P.	Journal of Arthroplasty	Functional outcomes and patient satisfaction in fast-track total knee arthroplasty: An Indian perspective	2022	68
4	Maniar, R.N.	Knee Surgery & Related Research	Effectiveness of intravenous and topical tranexamic acid in reducing blood loss in TKA: A randomized trial	2021	61
5	Mullaji, A.B.	The Knee	Coronal alignment and ligament balance in robotic-assisted total knee arthroplasty: A prospective study	2022	56

TKA = Total Knee Arthroplasty

### Language

The linguistic analysis of the TKA-related research output from India between 2021 and 2025 reveals a near-total dominance of the English language. Out of the 860 documents included in the final analysis, 859 (99.88%) were published in English. Only a single publication (0.12%) was identified in Spanish.

### Collaboration analysis

**Fig. 9:**
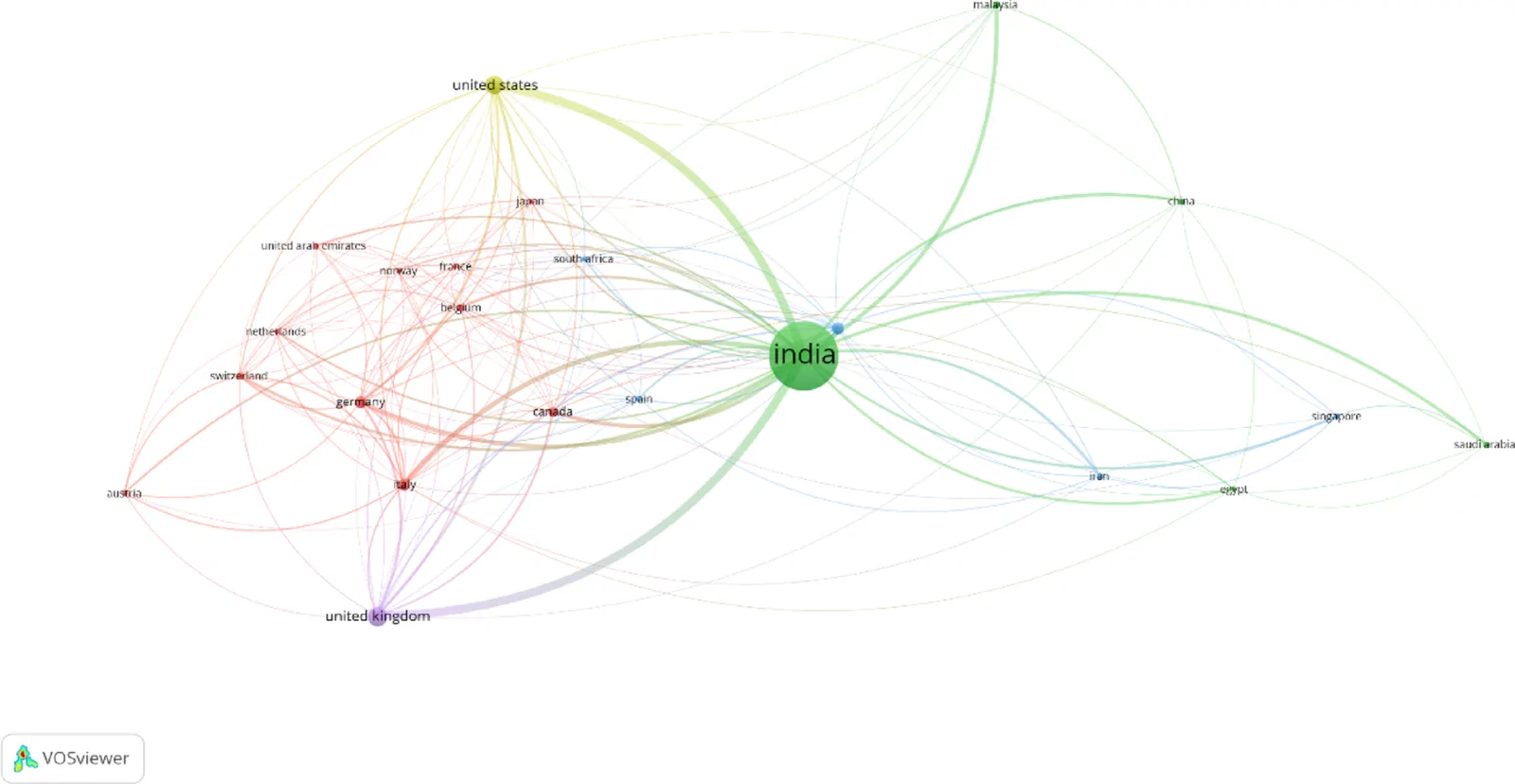
Network visualization of international country co-authorship in Indian TKA research generated using VOSviewer. Countries with a minimum of five publications were included in the analysis. Node size represents the number of publications contributed by each country, links indicate international co-authorship relationships, link thickness reflects the strength of collaboration (total link strength), and colours denote distinct collaboration clusters

Inter-institutional and inter-state collaborations increased over the five-year period. The international research output on TKA from India is supported by a diverse global network, with approximately 24.18% (*n* = 208) of all papers involving international co-authors. The United Kingdom (*n* = 71) and the United States (*n* = 64) are the primary collaborating partners, reflecting strong academic and clinical ties with Western institutions. European collaboration is also highly prominent, led by Italy (*n* = 31), Germany (*n* = 29), and Switzerland (*n* = 15), alongside significant partnerships with Australia (*n* = 27) and Canada (*n* = 18). Within the Asian and Middle Eastern regions, researchers have collaborated frequently with colleagues in Malaysia (*n* = 15), China (*n* = 12), and Saudi Arabia (*n* = 11). This wide geographical reach spanning North America, Europe, Southeast Asia, and Oceania demonstrate the global integration of the Indian arthroplasty community and its commitment to aligning with international surgical standards and technological innovations. Figure 9 depicts the visual representation of co-authorship countries in Indian TKA research in the past five years.

The analysis reveals that interdisciplinary and multidisciplinary collaborations are pivotal drivers of research in this field, appearing most prominently in the management of specialized conditions and the integration of advanced technologies. The data highlights a significant synergy between engineering and medical disciplines accounting for approximately 7.2% (*n* = 62) of the publications as evidenced by collaborative research between technical institutes and orthopedic centers on robotic-assisted arthroplasty, biomechanical modeling, and AI-driven surgical planning software. These interdisciplinary efforts extend to the implementation of Enhanced Recovery After Surgery (ERAS) and fast-track protocols, which require coordinated action across surgical, anesthetic, and nursing teams, as well as the development of global surgical guidelines during the COVID-19 pandemic. Collectively, these findings underscore that high-impact research in this domain is increasingly dependent on the convergence of clinical expertise, technological innovation, and integrated care pathways.

## Discussion

This bibliometric analysis provides a comprehensive overview of the evolution of TKA research in India over the past five years, a period marked by rapid clinical expansion, technological adoption, and increasing global engagement. Unlike previous bibliometric evaluations that examined broader arthroplasty literature over longer timeframes, the present study specifically focuses on TKA during the contemporary period (2021–2025), providing an updated assessment of current publication trends, institutional productivity, collaborative networks, and research priorities in India. By focusing on this recent timeframe, the present study captures contemporary research priorities that are most relevant to current practice, policy-making, and future innovation in Indian arthroplasty care.

### Evolution and growth trajectory in TKA research in India

The historical growth of TKA research in India, as evidenced by the 1,710 identified documents, reflects a transition from a nascent clinical discipline to a globally recognized research hub. The contrast between the “zero-publication” years of the early 1990s and the peak of 221 publications in 2025 highlights a maturation of the Indian orthopaedic ecosystem. Several factors contribute to this exponential surge. First, the acceleration phase (2016 onwards) coincides with the mass-market entry and clinical validation of robotic-assisted surgery and navigation systems in India. These technological advancements have not only improved surgical precision but have also provided a wealth of digital data that has fueled clinical trials and observational studies [27, 28]. Second, the institutionalization of research in both public centers (like AIIMS) and large private healthcare networks (like Apollo and Ganga Hospital) has created a sustainable pipeline for high-volume data collection [29, 30].

Nearly half of all Indian TKA publications have been produced in the last five years, indicating a marked acceleration in research activity and the growing contribution of Indian orthopaedic researchers to primary evidence generation [31, 32]. While the 2023 dip illustrates the vulnerability of elective surgical research to global health crises, the subsequent 75% growth by 2025 underscores a resilient recovery. This trajectory suggests that India is well-positioned to leverage its immense surgical volume to lead global discourse on TKA outcomes in the coming decade [28, 29, 32].

### Growth trajectory and contemporary relevance of the past five years

The identification of 860 Indian publications within a five-year window reflects a substantial and accelerating contribution to global TKA literature. The transient decline observed in 2023 may plausibly reflect disruptions in elective surgical services and academic activity during the post-pandemic recovery phase, a pattern reported across multiple surgical disciplines. The subsequent rebound and marked surge in 2024–2025 indicate a strong recovery and renewed academic momentum. Focusing on the past five years is particularly important in the Indian context, as this period coincides with widespread adoption of robotic-assisted TKA, enhanced recovery protocols, improved implant availability, and increased participation of private healthcare institutions in research [9, 33]. Consequently, the recent literature is more likely to reflect current surgical standards, patient expectations, and health-system realities than earlier decades [8–10].

This focused evaluation also complements previous bibliometric studies, including those by Vaishya et al., which primarily examined broader arthroplasty literature or combined hip and knee arthroplasty publications over extended periods. Restricting the analysis to the recent five-year period enables a contemporary assessment of research activity during an era characterized by increasing adoption of robotic-assisted surgery, digital health technologies, and post-pandemic recovery of elective orthopaedic services [19, 21, 22, 34].

### Global comparison: Where India stands and what is lacking

When compared to global benchmarks, the Indian TKA research landscape shows distinct characteristics. While the USA and UK lead in long-term survivorship data and multicentric registry-based studies [35, 36], and China and Japan dominate in biomechanical engineering and basic science research [34, 37, 38], India’s strength lies in clinical outcome reporting and “surgical technique” innovations [22, 39]. However, a critical gap remains. Unlike the UK’s National Joint Registry (NJR) or the Australian Orthopaedic Association National Joint Replacement Registry (AOANJRR), India lacks a unified, mandatory national registry that translates into high-impact publications [36, 40]. The current research landscape is hampered by fragmented data sources, where high-quality clinical information remains siloed within individual institutions rather than being integrated into a national database.

Furthermore, there is a significant over-representation of metropolitan private centers in the literature, which may not accurately reflect the surgical realities, patient demographics, or resource constraints of rural and public-sector healthcare settings. Currently, Indian research is heavily weighted toward short-to-mid-term clinical outcomes (2–5 years), resulting in critically limited long-term survivorship data [22, 31, 39, 41]. To bridge the gap with global leaders like the USA or Japan, Indian researchers must pivot toward long-term (10–15 year) survivorship data and large-scale cost-effectiveness analyses that account for the unique socio-economic diversity of the Indian patient population.

### Collaborative authorship and research evolution

The predominance of multi-authored publications, with an average of nearly five authors per paper, underscores the increasingly collaborative nature of TKA research in India. The dominance of mid-sized research teams suggests a balance between institutional collaboration and focused clinical leadership. The presence of larger author groups, including multi-institutional collaborations, reflects growing research networking and the conduct of more complex studies requiring diverse expertise. The rarity of single-author publications further indicates a shift away from isolated reporting toward team-based research, which is characteristic of mature scientific ecosystems. This trend aligns with global arthroplasty research patterns and supports the notion that Indian TKA research has progressed toward greater methodological rigor and shared academic responsibility [42, 43].

### Geographic concentration and regional disparities

State-wise analysis revealed a pronounced concentration of research output in metropolitan and academically established regions, particularly Maharashtra (*n* = 207) and Delhi (*n* = 168), followed by southern states such as Tamil Nadu (*n* = 114), Karnataka (*n* = 95), and Telangana (*n* = 68). These regions host a dense network of tertiary care hospitals, academic institutions, and high-volume arthroplasty centers, which naturally facilitates both clinical research and publication output. While the emergence of moderate contributions from several other states is encouraging, the limited output from northeastern and smaller states highlights persistent regional disparities. Addressing these imbalances is essential for generating nationally representative data and for ensuring that research findings are applicable across diverse healthcare settings in India. Capacity-building initiatives, multicentric registries, and collaborative networks may help bridge this gap [44, 45].

### Institutional leadership and the public–private research ecosystem

Institutional analysis demonstrates that both public academic centers and private healthcare institutions play a pivotal role in advancing TKA research in India. The leading contribution from AIIMS (*n* = 92) underscores the enduring importance of publicly funded academic institutions in driving high-quality clinical research. Simultaneously, the substantial output from large private hospital networks and specialty centers, such as the Apollo Hospitals group (*n* = 52), reflects the expanding role of the private sector in academic orthopaedics. This coexistence of public and private research leadership represents a distinctive strength of the Indian arthroplasty ecosystem [39]. High surgical volumes in private institutions, coupled with academic rigor in public centers, create complementary opportunities for innovation, large-scale data generation, and translational research [46, 47].

### Journals and international visibility

The concentration of publications in established national journals, particularly the Indian Journal of Orthopaedics (*n* = 102), highlights the importance of domestic platforms in disseminating context-specific research. Concurrently, the substantial presence of Indian studies in high-impact international journals such as the Journal of Arthroplasty (*n* = 44) and International Orthopaedics (*n* = 15) reflects increasing global recognition of Indian clinical data. The predominance of English-language publications (99.88%) further underscores the strategic alignment of Indian researchers with global scholarly standards, ensuring maximum international reach and citation potential. The appearance of technology-focused journals among the top publication outlets further reinforces the growing emphasis on robotics, navigation, and engineering integration in Indian TKA research [48, 49]. Additionally, the fact that over half of the research (52.7%) is disseminated via Open Access models reflects a commitment to clinical transparency and the democratization of surgical knowledge. This dual dissemination strategy: national relevance with international visibility strengthens the global positioning of Indian arthroplasty scholarship.

### Thematic evolution toward technology and multidisciplinary research

The thematic distribution of publications clearly indicates a transition from predominantly clinical outcome reporting toward a more multidisciplinary research paradigm. While orthopaedics and surgery remain the core domains (*n* = 603), the substantial contribution from engineering and technology (*n* = 98) suggests an increasing research interest in robotics, computer navigation, artificial intelligence, and implant material science within the retrieved literature [49, 50]. The keyword overlay visualization further demonstrated the temporal evolution of research themes, with earlier publications focusing predominantly on conventional clinical topics and more recent studies emphasizing artificial intelligence, robotic-assisted surgery, computer navigation, and precision alignment techniques. This temporal shift objectively supports the emergence of technology-driven research themes within Indian TKA literature.

Equally noteworthy is the growing body of rehabilitation-focused literature (*n* = 56), emphasizing functional outcomes, quality of life, and post-operative recovery. This shift reflects a broader, patient-centered approach to arthroplasty research that extends beyond surgical success to long-term functional restoration [51]. Beyond identifying emerging research themes, the network-based analyses provided additional insights into collaborative research patterns, thematic relationships, and influential contributors within Indian TKA research. The co-authorship and citation network analyses identified key collaborative groups and leading authors, highlighting the increasingly multidisciplinary and interconnected nature of the field. These findings are consistent with recent bibliometric studies that have emphasized the value of network-based analyses in understanding research evolution and collaboration patterns [23–25].

### Study types and research depth

The dominance of original research (*n* = 589) articles underscores strong primary research activity within India, supported by a meaningful volume of review articles (*n* = 101) that synthesize evolving evidence. The presence of book chapters, letters, and editorials suggests active scholarly dialogue and critical engagement within the orthopaedic community. Collectively, this distribution reflects a research environment that balances data generation with academic reflection and debate.

### Influence, citations, and knowledge leadership

The citation analysis reveals that the most influential Indian TKA publications are closely linked to emerging technologies, biomaterials, and robotic surgery [52–54]. The prominence of authors contributing both high-volume and high-impact work indicates sustained academic leadership within the field. By focusing specifically on original research papers, the ranking highlights primary clinical data, confirming that Indian-led clinical trials are gaining significant global traction [55, 56]. Importantly, several highly cited studies address topics with global relevance, reinforcing the contribution of Indian researchers to international arthroplasty discourse [50, 54, 57].

### International collaboration and global integration

Approximately 24.18% (*n* = 208) of Indian TKA publications involved international collaboration, with strong ties to the United Kingdom, the United States, and multiple European countries. Such collaborations enhance methodological quality, facilitate knowledge exchange, and improve the global applicability of research findings. The breadth of international partnerships observed in this study highlights India’s increasing integration into global arthroplasty research networks [42]. Furthermore, the synergy between medical and engineering disciplines (7.2%) underscores the multidisciplinarity required for modern surgical innovation.

### Limitations and potential bias

This study has several limitations that should be considered when interpreting the results. Primarily, the data was retrieved exclusively from the Scopus database, which may not capture the entirety of TKA research output from India, potentially omitting journals indexed only in other databases such as PubMed or Embase. It is crucial to note that these findings reflect published academic output and not the actual clinical volume of TKA procedures performed across the country. Therefore, the institutional and state-wise rankings indicate productivity within Scopus indexed literature rather than surgical throughput. Readers should not be misled into thinking the listed institutions are the only ones performing TKA; rather, they are the entities publishing frequently in indexed journals. The study relied on author-provided keywords indexed in Scopus, which may not fully capture all thematic relationships within the literature.

Furthermore, bibliometric data is subject to inherent affiliation and authorship ambiguities within the Scopus database, which may affect the precision of institutional tracking. Regarding impact metrics, high citation counts should not be exclusively equated with clinical superiority or guideline level evidence. There may be citation inflation due to the inclusion of review articles or the novelty of technology focused studies. Consequently, citation impact reflects academic visibility rather than direct clinical effectiveness.

Another important aspect is that the bibliometric indicators quantify productivity and visibility but do not assess the methodological quality, level of evidence, or clinical robustness of individual studies. High publication volume does not necessarily equate to high quality or practice changing evidence. Finally, due to the lack of a mandatory national arthroplasty registry in India, this analysis could not incorporate standardized long-term survivorship or revision data. This limitation prevents a direct comparison with registry driven global studies which provide the gold standard for clinical outcomes.

### Strategic insights and implications for future research

The focused assessment presented in this study demonstrates that Indian TKA research has entered a phase of accelerated growth and increasing sophistication during the contemporary era. By providing an updated benchmark of publication trends, institutional productivity, collaboration patterns, and thematic priorities, this work complements previous longitudinal bibliometric investigations and offers a current reference for future research planning. Future progress is heavily contingent on transitioning from predominantly single-center reporting to a robust, multicentric research ecosystem. Consistent with contemporary bibliometric insights, future work should move beyond the “bibliographic coupling” of established techniques and instead focus on the “frontier trends” of digital surgery, biomechanical joint loading optimization, and biological augmentation to remain globally competitive [17–19].

By strengthening national arthroplasty registries and fostering active collaborations between public academic centers and large private healthcare networks, researchers can generate high-impact, real-world evidence that accurately represents the pan-Indian patient population. Furthermore, it is essential to decentralize research activity; incentivizing universities and hospitals in underrepresented regions, such as the North-East and Central India, will address current geographic disparities and ensure that surgical standards and outcomes are validated across the country’s diverse socio-economic and regional landscapes. From a bibliometric perspective, future investigations could incorporate advanced network-based approaches such as bibliographic coupling, co-citation analysis, and thematic evolution mapping to provide deeper insights into the intellectual structure, knowledge evolution, and emerging research frontiers of Indian TKA research.

Strategically, India is uniquely positioned to lead global discourse in value-based healthcare through indigenous innovation and cost-effectiveness analyses tailored specifically to the Indian healthcare context. While global research leaders dominate in specific niches, India has a unique opportunity to provide a blueprint for other developing economies regarding the implementation of cost-effective robotic pathways and the long-term performance of domestic implants. This evolution requires a deeper synergy between medical centers and technical institutes to move beyond clinical observations toward fundamental innovations in AI-driven surgical planning, personalized alignment, and biomechanically informed recovery protocols [19]. By emphasizing high-quality comparative studies, long-term survivorship data (10–15 years), and biomechanical rigor, the Indian orthopaedic community can transition from being a high-volume contributor to a global policy-setter, establishing the next generation of international standards in TKA care.

## Conclusions

Indian research on TKA has demonstrated sustained growth, increased research productivity, thematic diversity, and global engagement over the past five years. The recent body of literature reflects a clear shift toward technology-driven, multidisciplinary, and collaborative research, with growing emphasis on robotics, advanced implant science, and optimized rehabilitation strategies. The peak publication counts in 2025 and the robust contribution of original research underscore a maturing academic ecosystem. Continued prioritization of multicentre collaboration, development of national arthroplasty registries, and inclusive research participation across diverse regions will be pivotal in shaping the next phase of TKA research and advancing clinical excellence in India.

## Supplementary Information

Below is the link to the electronic supplementary material.

Supplementary Material 1

Supplementary Material 2

## Data Availability

Not applicable.

## Abbreviations

TKA: Total knee arthroplasty
TKR: Total knee replacement
