# Supplementary material for: Research trends in total knee arthroplasty in India: a five-year bibliometric analysis
Source: Arch Orthop Trauma Surg. 2026 Oct 7;146(1):364. doi: 10.1007/s00402-026-06488-w (PMC13645959; doi:10.1007/s00402-026-06488-w)
Supplement: Supplementary file 1 [file 402_2026_6488_MOESM1_ESM.docx]

**Search Strategy**

**Research Trends in Total Knee Arthroplasty in India: A Five-Year Bibliometric Analysis**

|  | **Search string** | **Number of records retrieved** |
| --- | --- | --- |
| **Search** | Title, abstract, and keyword (“total knee arthroplasty" OR "TKA" OR "total knee replacement arthroplasty" OR "total knee joint replacement" OR "knee replacement total" OR "total knee replacement" OR "knee replacement*" OR "TKR" OR "knee arthroplasty total" OR "knee arthroplast*") | 1710 |
| **Filters** | Duration: 1 January 2021 to 30 December 2025 | 860 |
| **Filters: Country** | India | |
| **Date of search** | 1 January 2026 | |

Number of studies considered in the bibiometric analysis: 860
